# Tuning the magnetic properties of altermagnetic MnTe with pressure

**DOI:** 10.1063/4.0001052

**Published:** 2025-10-27

**Authors:** Edison P Carlisle, Benjamin A Frandsen

**Affiliations:** Brigham Young University, Provo, UT, USA

## Abstract

Hexagonal MnTe is an antiferromagnet with a Néel temperature of 307 K. Recently, it has attracted enormous interest as a leading example of the recently discovered altermagnet phase, as well as potential applications as a magnetically enhanced thermoelectric and as a platform for antiferromagnetic spintronics. MnTe has the largest spontaneous magnetovolume effect of any known antiferromagnet, implying strong coupling between the magnetic moment and volume. Here we use neutron scattering with in situ applied pressure to determine the effects of pressure on the magnetic properties of MnTe and further explore this magnetostructural coupling. We find that applying pressure significantly increases the Néel temperature but decreases the ordered magnetic moment, which we explain through the lens of the tight-binding model and supporting density functional theory calculations. These results show that the magnetic properties MnTe can be tuned through pressure, which could find application in the construction of devices and study of altermagnetic properties.

